# Genetic Diversity Analysis and Fingerprint Construction for 87 Passionfruit (*Passiflora* spp.) Germplasm Accessions on the Basis of SSR Fluorescence Markers

**DOI:** 10.3390/ijms251910815

**Published:** 2024-10-08

**Authors:** Fengchan Wu, Guojun Cai, Peiyu Xi, Yulin Guo, Meng Xu, Anding Li

**Affiliations:** 1 Guizhou Institute of Biology, Guizhou Academy of Sciences, Guiyang 550009, China; wfc08062024@163.com (F.W.); 18786707764@163.com (P.X.); 13511909661@163.com (Y.G.); 2 College of Environment & Ecology, Hunan Agricultural University, Changsha 410128, China; gzu_gjcai@163.com; 3 College of Forestry, Nanjing Forestry University, Nanjing 210037, China; xum@njfu.edu.cn

**Keywords:** *Passiflora*, SSR fluorescent markers, genetic diversity analysis, fingerprinting

## Abstract

A comprehensive genetic diversity analysis of 87 *Passiflora* germplasm accessions domesticated and cultivated for several years in the karst region of Guizhou, China, was conducted utilizing simple sequence repeat (SSR) fluorescent markers. These *Passiflora* species, renowned for their culinary and medicinal value, could bring significant economic and ecological benefits to the region. This study aimed to assess the genetic resources of these species and facilitate the selection of superior cultivars adapted to the karst environment. Our analysis revealed an abundance of SSR loci within the *Passiflora* transcriptome, with single-base repeats being the most prevalent type. Through rigorous primer screening and amplification, we successfully identified 27 SSR primer pairs exhibiting robust polymorphisms. Further interrogation at eight microsatellite loci revealed 68 alleles, underscoring the high level of genetic diversity present in the cultivated accessions. The average expected heterozygosity was 0.202, with the ssr18 locus exhibiting the highest value of 0.768, indicating significant genetic variation. The mean polymorphic information content (PIC) of 0.657 indicates the informativeness of these SSR markers. Comparative analyses of the cultivated and potential wild progenitors revealed distinct genetic variations among the different *Passiflora* types. Genetic structure and clustering analyses of the 87 accessions revealed seven distinct groups, suggesting gene flow and similarities among the resources. Notably, a DNA fingerprinting system was established using eight SSR primer pairs, effectively distinguishing the selected cultivars that had adapted to the karst mountainous region. This study not only deepens our understanding of *Passiflora* genetic resources in the karst environment but also provides a valuable reference for conservation, genetic improvement, and cultivar selection. The rich genetic diversity of the *Passiflora* germplasm underscores their potential for sustainable utilization in breeding programs aimed at enhancing the economic and ecological viability of these valuable plant species.

## 1. Introduction

The genus *Passiflora* is the largest genus within the expansive Passifloraceae family, encompassing more than 520 species globally. The vast majority of these species are native to the Americas, particularly countries such as Colombia, Brazil, Ecuador, and Peru, while a few are dispersed throughout other tropical and subtropical regions, such as Southeast Asia, Australia, and New Zealand [1,2,3]. The *Passiflora* species, commonly referred to as passionflower, have been widely cultivated in tropical and subtropical regions worldwide because of their multifaceted benefits, including edibility, medicinal properties, and ornamental appeal [4]. In terms of edibility, approximately 60 species of *Passiflora* are considered palatable, with notable examples including *P. edulis*, *P. edulis* f. *flavicarpa*, *P. ligularis*, and *P. quadrangularis.* In terms of medicinal importance, numerous *Passiflora* plants boast a rich history of use in traditional folk medicines in several American and European nations, where they have been employed as remedies for a diverse array of neurological conditions [5]. Active compounds extracted from the leaves, fruits, peels, and seeds of these plants exhibit a range of beneficial effects, including sedative, antioxidant, anti-inflammatory, anxiolytic, and anticancer effects [6,7,8].

Molecular markers such as AFLP, SSR, and SNP provide polymorphic information at the DNA level, demonstrating high accuracy and precision, particularly suitable for situations requiring the precise differentiation of germplasm resources and varieties. These molecular markers are not influenced by environmental factors and can stably reflect genetic variations, serving as essential tools for genetic analysis and variety improvement by breeders and researchers [9]. The use of simple sequence repeat (SSR) molecular marker technology, on the other hand, involves the amplification of targeted DNA fragments and the detection of products through electrophoresis. These markers consist of 1–6 nucleotide repeat units, and variations in the number of these repeat units contribute to the genetic diversity among the cultivars. SSR marker technology follows the rules of Mendelian codominant inheritance, exhibits excellent stability and repeatability, possesses abundant polymorphic loci, provides large amounts of information, and requires minimal DNA amounts. SSR technology has been extensively employed in various applications, such as DNA fingerprinting, kinship analysis, and genetic diversity studies. Its merits include codominant inheritance, abundant polymorphic loci, and low DNA requirements [10,11]. Specifically, SSR fluorescent labelling via capillary electrophoresis technology has been applied in plants, including pistachio [12], chestnut [13], upland cotton [14], plum [15], broomcorn millet [16,17], sweet potato [18], and apricot [19], primarily for analysing the genetic diversity of germplasm resources, constructing genetic maps, gene marking and mapping, cultivar identification, and parentage analysis. The International Union for the Protection of New Varieties of Plants (UPOV) recommends the combined use of SSR and SNP markers as the preferred method for constructing DNA fingerprints (UPOV 2007) [20].

Currently, the application of SSR molecular marker technology in the *Passiflora* species focuses primarily on genetic diversity analysis, kinship studies, and the conservation of germplasm resources. Santos et al. pioneered the identification of 669 SSR loci in a cultivated *Passiflora* species via the BAC-end method [21]. Araya et al. subsequently leveraged partial sequences from the genome sequencing of cultivated *Passiflora* species to develop 816 pairs of SSR primers targeting functional genomic regions. Among these, 57 SSR primer pairs were selected and validated in 79 samples of both wild and cultivated *Passiflora* species, revealing polymorphisms in 42 primer pairs [22]. Furthermore, da Costa et al. utilized cDNA sequences from suppression subtractive hybridization (SSH) libraries to devise 42 SSR primers, with 34 demonstrating high transferability between yellow/purple-fruited *Passiflora* and *Passiflora coccinea* [23]. Research has shown that the escaped habitats of *Passiflora* in karst peak-cluster depression regions are complex and diverse, where the *Passiflora* species coexist with companion species in a mutually beneficial symbiosis, adapting well to the environment while exhibiting rapid growth and high biomass production. In Guizhou’s karst region, *Passiflora* cultivation has emerged as a local industry. The growth of *Passiflora* in rocky desertification areas can improve soil porosity, increase aeration and water permeability, lower ambient temperatures, and increase humidity, resulting in significant cooling and humidifying effects during hot weather, thereby contributing positively to the ecosystem [24]. Selecting and breeding suitable *Passiflora* varieties for karst regions can provide both economic and ecological benefits. This study employed SSR fluorescent labeling via capillary electrophoresis technology to conduct a genetic diversity analysis of 87 *Passiflora* germplasm accessions domesticated and cultivated over several years in Guizhou’s karst region and Xishuangbanna, China. By leveraging the SSR markers developed from transcriptome sequencing, we aimed to elucidate the kinship relationships, population genetic structures, and genetic backgrounds of diverse *Passiflora* germplasm resources. This work provides a solid foundation for the selection, breeding, application, preservation, and molecular marker-assisted breeding of suitable *Passiflora* varieties in the karst regions.

## 2. Results

### 2.1. Characterization of SSR Loci in the Passionfruit Transcriptome

Through an analysis of the passionfruit transcriptome, the transcriptome was sequenced from the purple-fruited *Passiflora* from the Guizhou Province; we identified 27,303 SSR loci within 86,880 unigenes, exhibiting a frequency of 31.43%, with an average of one SSR locus per 4.6 kb. Notably, 4517 unigenes contained more than one SSR locus (Table 1). The SSR repertoire in the passionfruit transcriptome was diverse, encompassing all repeat types from mono- to hexanucleotide repeats, albeit with significant variations in abundance among the different repeat types. Mononucleotide repeats were the most abundant, accounting for 63.7% of all SSR loci, with A/T repeats being the dominant type. Dinucleotide repeats constituted 21.0% of the total, dominated by AG/CT repeats. Trinucleotide repeats constituted 14.0% of the SSR loci, with AAG/CTT repeats being prevalent. Tetranucleotide repeats accounted for 0.9% of the total, with AAAT/ATTT repeats being the most numerous. Pentanucleotide and hexanucleotide repeats were less frequent, with proportions of 0.3% and 0.1%, respectively.

The repeat number is a crucial factor influencing SSR polymorphism. The repeat numbers of SSRs in the passionfruit transcriptome ranged from 5 to 24, with 10 repeats being the most abundant. Among the different repeat types, mononucleotide repeats were predominant (10 repeats), followed by dinucleotide repeats (6 repeats), and trinucleotide, tetranucleotide, and pentanucleotide repeats (5 repeats). Interestingly, hexanucleotide repeats showed a higher abundance (6 repeats). The number of SSR loci for mononucleotide to pentanucleotide repeats decreased with increasing repeat number, whereas for hexanucleotide repeats, the repeat number increased from 5 to 6.

### 2.2. Screening of SSR Primers

A total of 50 loci were randomly selected for primer design, and six randomly chosen passionfruit samples were used to screen the 50 pairs of SSR primers. As a result, 27 primer pairs were identified that exhibited a distinct and robust amplification of major bands (Table 2). Among these primer pairs, eight displayed stable amplification with clear major bands and exhibited good polymorphism. The primer numbers for these eight selected primers were SSR1, SSR2, SSR3, SSR18, SSR30, SSR32, SSR34, and SSR39, and the amplification patterns of the two samples in this study using eight core primers are shown in Figure S1.

### 2.3. SSR Polymorphic Variation

In the genetic assessment of 87 *Passiflora* germplasm resources utilizing eight core primers, a total of 68 alleles were detected, ranging from 4 alleles for SSR30 to 15 alleles for SSR39, with an average of 8.625 alleles per primer. Each sample contributed either two identical (monomorphic, single peak) or distinct (polymorphic, double peak) alleles at each locus. The effective number of alleles (Ne) varied from 1.977 for SSR34 to 4.724 for SSR18, with an average of 3.100. The observed heterozygosity (Ho) ranged from 0.079 for SSR2 to 0.449 for SSR18, with a mean value of 0.235. In contrast, the expected heterozygosity (He) ranged from 0.494 for SSR34 to 0.788 for SSR18, with an average value of 0.650. The Shannon diversity index (I), which quantifies the uncertainty associated with predicting the identity of an allele from a randomly chosen individual, ranged from 1.021 for SSR34 to 1.786 for SSR32, with a mean of 1.410. Notably, the polymorphic information content (PIC), a measure of the informativeness of a genetic marker, exceeded 0.250 for all the primers, ranging from 0.460 for SSR34 to 0.762 for SSR18, with an average of 0.614 (Table 3). These results demonstrate the high level of genetic diversity and potential utility of the selected SSR markers in genetic studies of *Passiflora* species.

### 2.4. Analysis of Genetic Diversity among Passiflora Germplasm Resources

This study analysed 87 germplasm resources belonging to 22 species and their cultivars within the genus *Passiflora*. The range of the average number of alleles (Na) per population varied from 0.750 in *P. xishuangbannaensis* to 2.750 in *P. edulis*. Similarly, the average effective number of alleles (Ne) also showed a significant variation, ranging from 0.750 in *P. xishuangbannaensis* to 1.880 in *P. edulis*. Notably, both *P. miniata* Xishuangbanna Red and *P. serrulata* presented a value of zero for average expected heterozygosity (He), Shannon’s diversity index (I), and observed heterozygosity (Ho). In contrast, the remaining 22 species and their cultivars presented a broader range of genetic diversity indices, with the He values ranging from 0.055 for *P. miniata* to 0.436 for *P. edulis*, the I values varying from 0.087 for *P. caerulea* to 0.701 for *P. edulis*, and the Ho values ranging from 0.063 for *P. miniata* to 0.563 for *P.* ‘*Lady Margaret*’ (Table 4).

### 2.5. Genetic Structures of Passiflora Germplasm Resources

**Figure 1:**
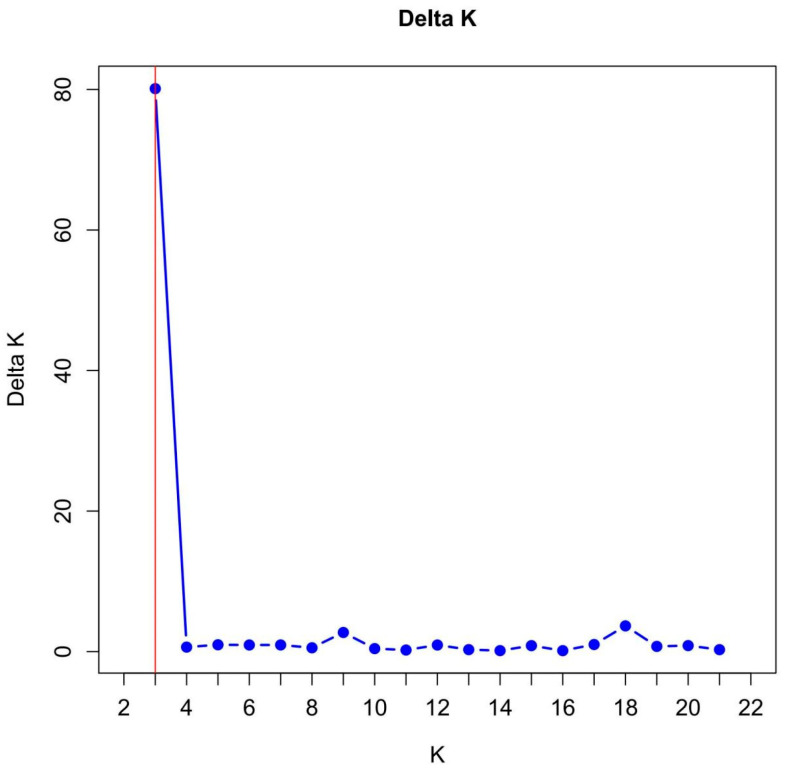
Graphical depiction of the relationship between K and Δk.

The genetic structure of 87 passionfruit (*Passiflora* spp.) germplasm accessions was analysed on the basis of SSR molecular marker data. The analysis was performed by setting the number of clusters (K) ranging from 1 to 22, with 15 repetitions for each K value. The optimal K value was determined using the ΔK, which peaked at K = 3 (Figure 1). Notably, there was a distinct increase in ΔK at K = 9, suggesting the incorporation of a new subpopulation within the tested materials, indicating that K = 9 represents a crucial subdivision point (Figure 1).

**Figure 2:**
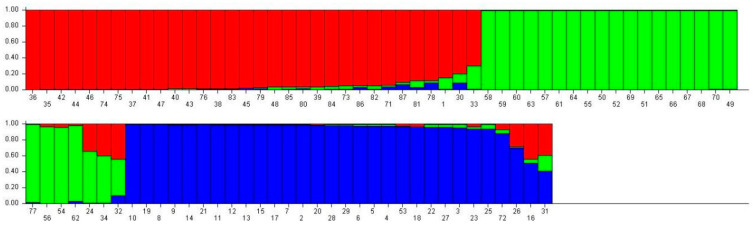
Population genetic structure of 87 *Passiflora* germplasm accessions (K = 3).

When K = 3, the 87 accessions were divided into three distinct groups (Figure 2), implying three potential genetic origins. The red group consisted primarily of species such as *P. amethystina*, *P. foetida*, *P. quadrangularis*, *P. caerulea*, *P. incarnata × P. laurifolia*, *P. incarnata*, *P. suberosa*, *P. violacea* ‘*victoria*’, *P. morifolia*, *P. yucatanensis*, *P. altebilobata*, *P. trifasciata*, and *P.* ‘*Lady Margaret*’. The green group was dominated by *P. miniata*, *P. miniata* Xishuangbanna Red, *P. serrulata*, *P. ligularis*, *P. miniata × P. serrulata*, and *P. serrulata.* Finally, the blue group was composed mainly of cultivated varieties of *P. edulis*, *P. edulis* f. *flavicarpa*, and their hybrids.

**Figure 3:**
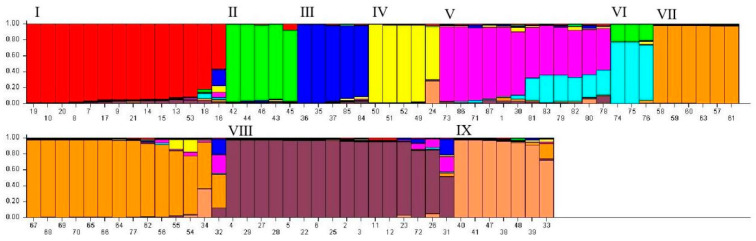
Population genetic structure of 87 *Passiflora* germplasm accessions (K = 9).

At K = 9, a more detailed subdivision was observed, with the 87 accessions classified into nine groups (Figure 3), suggesting nine potential genetic origins. Groups 1 and 8 were dominated by *P. edulis*, *P. edulis* f. *flavicarpa*, and their hybrids. Group 2 contained only *P. foetida*, whereas Group 3 was characterized by *P. hystina*, *P. caerulea*, and *P. violacea* ‘*victoria*’. Group 4 was predominantly *P. ligularis* and *P. edulis* f. *flavicarpa* “*mi zhuan*”, with the latter’s primary genetic contribution coming from Groups 4 and 8. Group 5 was composed of *P. morifolia*, *P. yucatanensis*, *P. alitebilobata*, *P. trifiata*, and *P. suberosa*, with *P. suberosa*’s major gene flow originating from Groups 5 and 6. Group 6 exclusively consisted of *P. quadrangularis*, with gene flow from Groups 2 and 6. Group 7 was dominated by *P. miniata, P. miniata × P. serrulata*, *P. miniata* Xishuangbanna Red, *P. serrulata*, *P.* ‘*Lady Margaret*’, and *P. xishuangbannaensis*, with the latter exhibiting the most complex genetic admixture from Groups 3, 5, 7, and 8. Finally, Group 9 was mainly composed of *P. incarnata × P. laurifolia*, *P. incarnata*, and *P.* ‘*Lady Margaret*’. The mixed composition of species within each group suggests that these varieties do not exhibit significant differences in their most primitive population classifications. To better understand the evolutionary patterns among different populations, tracing the origins of some varieties back to their more primitive habitats is necessary.

In the STRUCTURE population structure analysis, we assessed the association of individuals with different genetic clusters based on their Q values, which represent the probability of an individual belonging to a particular cluster. A Q value ≥0.6 was considered indicative of a significant genetic contribution from that cluster. For the 87 *Passiflora* germplasm accessions, the distribution of Q values across clusters is presented in Table 4. Results show that 79 accessions (90.8% of the total) had Q values >0.6 in at least one cluster, indicating strong genetic affiliations with at least one cluster. Furthermore, 65.5% and 56.3% of the accessions had Q values ≥0.8 and ≥0.9, respectively, in at least one cluster, reflecting their close genetic relationships with specific clusters. Nevertheless, all the accessions likely contain genetic variations from the other clusters to some extent, highlighting the genetic diversity within the germplasm collection (Table 5).

### 2.6. Cluster Analysis of Passiflora Germplasm Resources

**Figure 4:**
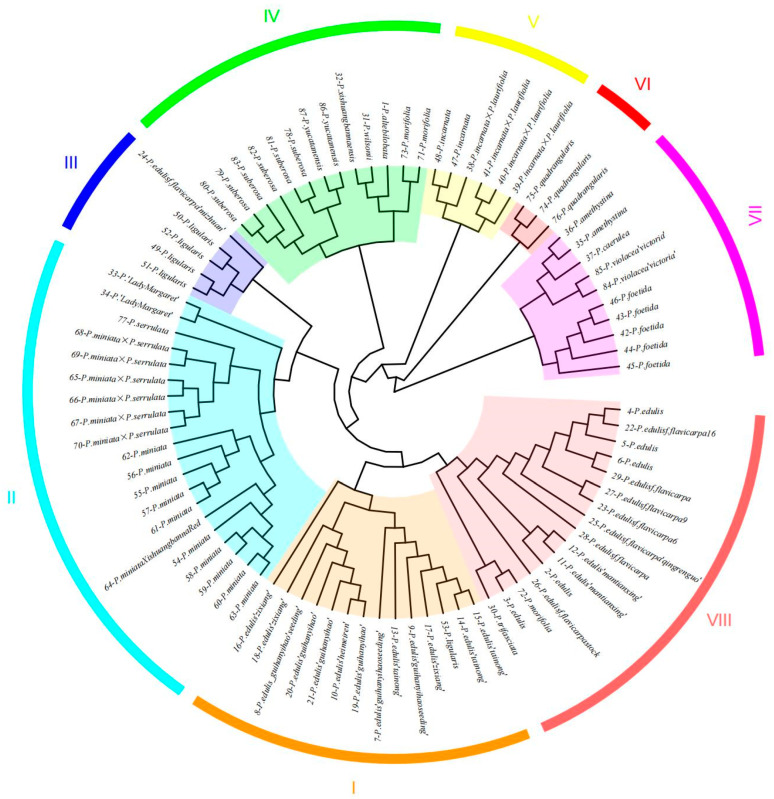
UPGMA clustering dendrogram of 87 *Passiflora* germplasm accessions.

A cluster analysis was conducted on 87 *Passiflora* germplasm resources on the basis of Nei’s genetic distance. The results indicated that eight pairs of core primers were able to distinguish these 87 *Passiflora* germplasm resources, which were subsequently grouped into eight distinct clusters (Figure 4). Cluster 1 consisted primarily of cultivated varieties of *P. edulis*. Cluster 2 was composed mainly of *P. miniata* × *P. serrulata*, *P. miniata* Xishuangbanna Red, *P.* ‘*Lady Margaret*’, and *P. serrulata*. Cluster 3 was dominated by *P. edulis* f. *flavicarpa* ‘*mizhuan*’ and *P. ligularis*. Cluster 4 included *P. yucatanensis*, *P. morifolia*, *P. altebilobata*, *P. wilsonii*, and *P. xishuangbannaensis*. Cluster 5 was composed primarily of *P. incarnata* × *P. laurifolia* and *P. incarnata*. Cluster 6 was composed exclusively of *P. quadrangularis*. Cluster 7 included *P. caerulea*, *P. amethystina*, *P. foetida*, and *P. violacea* ‘*victoria*’. Finally, Cluster 8 was composed primarily of cultivated varieties of *P. edulis*, *P. edulis* f. *flavicarpa*, and *P. trifasciata*.

### 2.7. Construction of Fingerprint Profiles for Passiflora Germplasm Resources

The eight primer pairs were ranked in descending order on the basis of their PIC values presented in Table 3. The resulting order was as follows: ssr18, ssr3, ssr32, ssr2, ssr39, ssr30, ssr1, and ssr34. The corresponding codes for each fragment amplified by these primers are provided in Table 6. By utilizing the primer order and fragment sizes of the amplified bands (where ‘0’ signifies the absence of a discernible peak), alphabetically arranged fingerprint profiles were constructed for each germplasm resource (Table 7). These fingerprint profiles serve as robust tools for identifying and differentiating *Passiflora* germplasm resources, meeting the standards for scientific reporting in international journals.

## 3. Discussion

### 3.1. Characterization of Passiflora SSR Loci and Primer Screening

In this study, 86,880 unigenes were identified from the *Passiflora* transcriptome, resulting in the identification of 27,303 SSR loci, with an SSR frequency of 31.43%. Research on microsatellite markers developed for the *Passiflora* species has often detected low polymorphism, with SSR frequencies ranging from 15% to 24.7% [21]. The frequency and occurrence of SSRs are influenced by factors such as species, taxonomic groups, SSR databases and development tools, and SSR search criteria [23,25,26].

In many plants, dinucleotide and trinucleotide repeats are the primary repeat types, albeit with differing dominant motifs [27,28]. In the present study, mononucleotide, dinucleotide, and trinucleotide repeats were predominant in the *Passiflora* transcriptome, accounting for 63.72%, 20.40%, and 14.28% of the total SSRs, respectively. In contrast, tetranucleotide, pentanucleotide, and hexanucleotide repeats constituted a smaller proportion (1.6%). Among the SSR motifs in *Passiflora*, A/T was the most abundant among the mononucleotide repeats, AG/CT dominated among the dinucleotide repeats, and AAG/CTT was the most prevalent among the trinucleotide repeats, all of which are consistent with the observation by Meng et al., who reported that AAG/CTT is a common motif in dicotyledonous plants. Additionally, the scarcity of CG/GC, a common feature in most dicotyledonous plants, was also observed in *Passiflora*, which presented the lowest CG/GC content [29].

The polymorphism level of the SSR molecular markers is a crucial criterion for assessing their usability, and SSR length is a major factor influencing polymorphism. SSRs with lengths ≥20 bp exhibit high polymorphism, whereas those between 12 and 20 bp in length display moderate polymorphism, and those shorter than 12 bp have extremely low polymorphism [30]. In this study, 50 SSR primers were designed on the basis of randomly selected loci, and six *Passiflora* samples were used to screen these 50 primer pairs. A total of 27 primer pairs produced clear and distinct primary bands, among which 8 were further selected for their stable amplification, clear bands, and polymorphism. Studies have shown that microsatellite markers developed for *Passiflora edulis* var. *flavicarpa* can be utilized for the genetic analysis of other *Passiflora* species. Silva et al. examined the transferability of 31 previously designed SSR primers between the wild and cultivated *Passiflora* species and reported high transferability for several SSR markers, indicating the feasibility of establishing universal SSR markers for both wild and cultivated *Passiflora* species. These markers can be applied to studies on the genetic diversity, identification, and exploitation of *Passiflora* germplasm resources, providing polymorphic molecular markers for further investigations of the genetic variation patterns of *Passiflora* germplasm resources [31].

### 3.2. Analysis of Genetic Diversity in Passiflora Germplasm Resources

Genetic diversity serves as a crucial indicator in the study of plant adaptability to environmental changes, with low genetic diversity leading to species decline in nature [32,33]. The similarity between the observed and expected heterozygosity values can also indicate the level of genetic diversity within a population, with more similar values indicating a greater genetic diversity [13]. Among the population genetic parameters, He is commonly used to measure the genetic diversity of a group. A higher He value reflects lower genetic uniformity and richer genetic diversity. In this study, the average He value of the samples of the 87 *Passiflora* species was 0.202, with the highest He value of 0.768 observed at the ssr18 locus, indicating substantial genetic diversity among the tested *Passiflora* samples.

The Ne value is a significant indicator of genetic variation within a population. An Ne value close to the absolute number of alleles detected suggests a uniform distribution of alleles, whereas a large discrepancy indicates uneven distribution [34,35,36]. In this study, the number of alleles and Ne detected by the eight core primers varied among the 87 *Passiflora* germplasm resources, indicating an uneven distribution of alleles among the tested samples.

The PIC is used to quantify the degree of polymorphism at a locus. A PIC value greater than 0.5 indicates a high degree of polymorphism, whereas values between 0.25 and 0.50 suggest reasonable polymorphism, and values below 0.25 indicate low polymorphism [37]. Silva et al. utilized 31 previously designed SSR primers to study the transferability of SSR markers between wild and cultivated *Passiflora* species, reporting PIC values ranging from 0.26 to 0.70, with an average of 0.46 [31].

Previous studies have employed SSR markers to assess genetic variation in 51 Colombian commercial yellow *Passiflora* accessions (102 individuals), reporting a PIC of 0.74 and Ho and He values of 0.52 and 0.78, respectively [38]. Using RAPD and ISSR markers, studies on 20 passionfruit resources from different provinces in Southern Vietnam reported PIC values ranging from 0.85 to 0.88 [39]. Analysis of 70 purple *Passiflora* accessions via AFLP and SSR markers revealed low genetic diversity [40]. A study on 127 *Passiflora* species samples from Brazil and Colombia, including 85 commercial *P. edulis* samples and 42 samples from 13 wild species, reported average Ho and He values of 0.43 (ranging from 0.01 to 0.77) and 0.50 (ranging from 0.01 to 0.86), respectively, for *P. edulis* [41]. A study of 79 *Passiflora* species with 18 SSRs yielded an average PIC value of 0.60, ranging from 0.46 to 0.77 [22].

Collectively, these findings demonstrate the effectiveness of SSR markers in assessing genetic diversity and characterizing *Passiflora* germplasm resources, contributing to efforts to harness the full potential of *Passiflora* for agricultural, medicinal, and ornamental applications.

### 3.3. Genetic Structure and Cluster Analysis of Passiflora Germplasm Resources

Genetic structure analysis and cluster analysis were performed on 87 *Passiflora* germplasm resources. The results revealed that these resources could be categorized into eight to nine distinct groups. Both analyses categorized the cultivated *Passiflora* species *P. edulis*, *P. edulis* f. *flavicarpa*, and their hybrids into two main groups, one primarily comprising the yellow-skinned varieties and the other consisting primarily of the purple-skinned varieties. This finding aligns with those of the previous studies on Fujian passionfruit [42] and Brazilian passionfruit [22]. Notably, the genetic variation among passionfruit cultivars and lines was substantial, with no clear correlation observed between the geographical origins of the tested samples and their genetic differences.

Species and varieties with red flowers, such as *P. miniata*, *P. miniata* Xishuangbanna Red, and *P.*‘*Lady Margaret*’ were grouped into one cluster, whereas those with purple flowers, including *P. caerulea*, *P. amethystina*, and *P. violacea* ‘*victoria*’, constituted another distinct cluster. Additionally, the hybrid *P. incarnata* × *P. laurifolia* and the species *P. incarnata* itself formed a separate group, which is consistent with the findings of Anderson et al. [34]. The introduction of *P. incarnata* (or a related species) to the karst regions for cultivation has demonstrated strong stress tolerance, and studies have suggested that it is one of the species most similar to *P. edulis* in terms of valuable traits such as cold tolerance and phenotypic diversity (e.g., flowering time, flower colour, fruit size, and fertility), despite its inferior fruit quality.

In the cluster analysis of this study, most species clustered with those belonging to the same subgenus. Specifically, *P. morifolia* and *P. trifasciata*, both belonging to the Decaloba subgenus, were grouped with *P. edulis* and *P. edulis* f. *flavicarpa* (of the *Passiflora* subgenus). This outcome may be attributed to the limited number of molecular marker primers used in this study, which failed to distinguish *P. morifolia* and *P. trifasciata* from other species. *P.* ‘*Lady Margaret*’ is the fanciful name given to an F1 progeny resulting from an interspecific artificial cross between *P. coccinea* and *P. incarnata* in 1991. Phenotypically, *P.* ‘*Lady Margaret*’ exhibits greater similarity to *P. coccinea* [43]. The failure of *P. ‘Lady Margaret’* to cluster with *P. incarnata* could stem from genetic differences; despite the hybridization between *P. coccinea* and *P. incarnata*, the hybrid offspring may have inherited diverse genetic traits from both parents. These traits manifest as distinct amplification patterns or polymorphic loci in SSR molecular markers, thereby contributing to a genetic divergence between the hybrid and either parent. This divergence is sufficient to prevent *P.* ‘*Lady Margaret*’ from grouping with *P. incarnata* in the cluster analysis. Additionally, genetic recombination, a crucial mechanism in biological evolution, may have occurred during the hybridization process. In the cross between *P. coccinea* and *P. incarnata*, genetic recombination could have led to novel genetic combinations in the offspring. These novel combinations might significantly differentiate the hybrid from either parent in terms of SSR molecular markers. Similarly, in the study by Anderson et al., *P. incarnata* × *P. edulis* and *P. edulis* × *P. incarnata* did not cluster with *P. incarnata* and displayed relatively distant genetic relationships [44].

### 3.4. Construction of DNA Fingerprints for Passiflora Germplasm Resources

The construction of DNA fingerprints, characterized by multiple loci, high variability, and simple yet stable inheritance, is considered the simplest and most effective approach for cultivar identification. The higher the PIC value of a primer is, the more capable the primer is of distinguishing cultivar-specific traits, making it suitable for use as a core primer in constructing DNA fingerprinting profiles [37]. In this study, eight pairs of SSR primers were employed to establish the DNA fingerprinting profiles for 87 *Passiflora* germplasm resources, encompassing 22 species (cultivars) within the genus. Notably, these eight SSR primers were fully capable of differentiating the 22 species (cultivars), and the constructed fingerprinting profiles also discriminated between some species (cultivars) that have been introduced and cultivated for extended periods in the karst mountains of Guizhou. Traditional methods of cultivar identification on the basis of phenotypic traits are often influenced by a combination of environmental factors. In contrast, DNA fingerprinting directly reveals the genetic differences among the cultivars at the DNA level, offering greater precision, polymorphism, and stability than phenotype-based identification does, and it is unaffected by environmental conditions or the plant’s growth status. These findings provide a direct and effective theoretical foundation for cultivar improvement and conservation [45].

## 4. Materials and Methods

### 4.1. Experimental Materials

The experimental materials consisted of 87 *Passiflora* germplasm resources that have been cultivated and domesticated for many years in the *Passiflora* Germplasm Nursery in Kedu Town, Pingtang County, Guizhou Province (25°44′ N, 106°48′ E, 884 m above sea level), and the Xishuangbanna Tropical Botanical Garden, Chinese Academy of Sciences (21°56′ N, 101°16′ E, 570 m above sea level). Table S1 presents a summary of these materials. For transcriptome sequencing, the material of interest was purple-fruited *Passiflora* from Pingtang County, Guizhou Province. The aboveground parts (stems, leaves, flowers, and fruits) of three individual plants were harvested, immediately frozen on dry ice, and transported for storage in an ultralow temperature freezer at −80 °C. RNA was extracted separately from stems, leaves, flowers, and fruits, and then equal amounts were pooled for subsequent transcriptome sequencing.

### 4.2. SSR Locus Mining and Primer Design

The SSR loci within the *Passiflora* transcriptome data were identified and analysed using MISA v2.1 (http://pgrc.ipk-gatersleben.de/misa/misa.html (accessed on 1 October 2020)) with the following parameters: minimum repeat numbers of 8, 5, 4, 3, 2, and 2 for mono-, di-, tri-, tetra-, penta-, and hexanucleotide motifs, respectively. Fifty SSR loci were randomly selected, and primers were designed using Primer5.0. The primers were synthesized by GenScript Biotech Corporation (Nanjing, China), dried to powder, diluted to 10 μmol/L, and stored at −20 °C for later use.

### 4.3. Extraction and Analysis of DNA from Passiflora

Genomic DNA was extracted from *Passiflora* samples via the BioTeke Plant Genomic DNA Extraction Kit. The concentration and purity of the DNA were measured via a Thermo Scientific NanoDrop 2000C spectrophotometer (Thermo Scientific, Waltham, MA, USA), and the quality of the DNA was preliminarily assessed on the basis of the OD values obtained. The DNA was diluted to a concentration of 30 ng/μL and stored at 4 °C for future use.

### 4.4. PCR System and Program Setup

The PCR mixture had a total volume of 10 μL, containing 1 μL of 10 × buffer, 0.4 μL of 25 mmol/L MgCl2, 1 μL of 2.5 mmol/L dNTPs, 0.3 μL each of 0.2 μmol/L forward and reverse SSR primers, 0.12 μL of Taq DNA polymerase (TAKARA), 1.5 μL of DNA template, and 5.38 μL of ddH_2_O. The PCR cycling conditions were as follows: initial denaturation at 94 °C for 5 min, followed by 30 cycles of denaturation at 94 °C for 30 s, annealing at 55–58 °C for 30 s, and extension at 72 °C for 30 s, with a final extension at 72 °C for 3 min.

### 4.5. Polyacrylamide Gel Electrophoresis

The PCR amplicon lengths were determined via 8% polyacrylamide gels. Electrophoresis was performed in a DYCZ-30A vertical electrophoresis tank (Beijing Liuyi Instrument Factory, Beijing, China) with a 50 bp marker as the standard molecular weight marker. Electrophoresis was conducted at 120 V and 350 mA for 120 min. The PCR products were sent to Sangon Biotech (Shanghai) Co., Ltd. Shanghai, China, for capillary electrophoresis analysis via an ABI 3730XL DNA Analyser. The internal size standard used was GS-500 LIZ.

### 4.6. Data Statistics and Analysis

#### 4.6.1. Polymorphism Analysis of Microsatellite Loci

The polymorphisms of the eight developed microsatellite loci were evaluated via GenAlEx 6.51b2 [46]. Key polymorphism indices, including the number of alleles (Na), the number of effective alleles (Ne), expected heterozygosity (He), observed heterozygosity (Ho), and Shannon’s diversity index (I), were obtained.

#### 4.6.2. Analysis of Genetic Variation within Populations

Genetic variation within the populations was also analysed via GenAlEx 6.51b2 [46]. Population genetic diversity, which measures the allelic and genotypic composition of a given population, is characterized by the Na, Ne, He, Ho, and I values. The polymorphism information content (PIC) was calculated via PowerMarker 3.25. Population structure was analysed with STRUCTURE 2.2.4 [29], employing the admixture model and allele frequency correlation model.

#### 4.6.3. Analysis of Population Genetic Structure and Clustering

A genetic dendrogram was constructed in MEGA 6.06 [28] on the basis of pairwise Nei’s standard genetic distances calculated via PowerMarker 3.25. A clustering heatmap was generated using R language code to visualise the clustering of the 87 *Passiflora* samples. STRUCTURE v2.3.4 was used to infer the population structure, employing the admixture model and allele frequency correlation model with 106 iterations of the Monte Carlo Markov chain (MCMC) algorithm. We tested K values ranging from 1 to 22, with 15 runs for each K. The optimal K value (number of subpopulations) was determined via the ΔK method using STRUCTURE HARVESTER (https://lmme.ac.cn/StructureSelector/ (accessed on 29 May 2024)), where the highest ΔK indicates the best-fit number of subpopulations.

#### 4.6.4. Selection of Core Primers and Construction of Fingerprint Maps

Core primers were selected on the basis of their PIC to establish fingerprint maps. Fragments amplified by different primers were numbered according to their length. A two-letter code represents each primer, and “0” indicates the absence of a clear band for that primer in a particular variety. On the basis of the order of the primers and the sizes of the amplified fragments, an alphabetically arranged fingerprint map was constructed for each genotype.

## 5. Conclusions

This study evaluated the genetic diversity of the *Passiflora* species which has been domesticated and cultivated for several years in the karst region of Guizhou, China, providing crucial theoretical and technical support for the classification, collection, conservation, elite breeding, and molecular marker-assisted breeding of *Passiflora* genetic resources. Through primer screening and the genetic diversity analysis of the SSR loci, a series of stable and highly polymorphic SSR primers were developed, and an in-depth investigation of the genetic structure of *Passiflora* species was conducted. The results revealed a wealth of genetic diversity and distinct genetic structures among the *Passiflora* resources. There was a certain degree of gene flow among the different populations, while some populations presented high genetic differentiation. Fingerprint maps were constructed to distinguish selected species (cultivars) that have been introduced and cultivated for years in the karst mountains. Genetic differences and relationships among the different germplasm resources were identified. These classifications offer important references and guidance for the conservation, utilization, and elite breeding of *Passiflora* genetic resources. This study provided in-depth insights into the genetic diversity, genetic structure, and phylogenetic relationships of *Passiflora* species, providing solid scientific evidence and technical support for the conservation and utilization of their germplasm resources. Future research could further expand the sample range, delve deeper into genetic information, and conduct comprehensive analyses incorporating phenotypic data to elucidate the genetic characteristics and evolutionary history of *Passiflora* species, thereby offering deeper insights and guidance for the conservation and utilization of their germplasm resources.

## Figures and Tables

**Table 1:** SSR Locus information from the passionfruit transcriptome.

Repeats	5	6	7	8	9	10–15	16–20	>21	Total
Mono-motif									(17,401)
A/T						13,911	2780	579	17,270
C/G						93	3	35	131
Din-motif									(5738)
AC/GT		256	148	113	44	80			641
AG/CT		1110	639	465	446	454	1		3115
AT/AT		615	423	313	284	336			1971
CG/CG		11							11
Tri-motif									(3808)
AAC/GTT	175	35	31	2		1			244
AAG/CTT	589	262	148	7					1006
AAT/ATT	157	72	57	8					294
ACC/GGT	221	87	65	9	1				378
ACG/CGT	55	37	20	11					123
ACT/AGT	31	14	4	2					51
AGC/CTG	290	128	45	4					467
AGG/CCT	399	145	123	3					670
ATC/ATG	270	98	65	6					439
CCG/CGG	89	31	13	3					136
Tetra-motif	216	24	6						249
Penta-motif	57	4	7		1				69
Hexa-motif	7	23	4	2		2			38
Total number of unigenes examined	86,880								
Total size of examined sequences (bp)									125,855,003
Total number of identified SSRs									27,303
Number of SSR containing unigenes									21,578
Number of unigenes containing more than 1 SSR									4517
Number of SSRs present in compound formation									1427

**Table 2:** The 27 SSR primers that amplified distinct major bands.

Name	SSR Motif	Forward (5′-3′)	Reverse (5′-3′)
SSR1	(GGAGTT)6	GGAGGAGGGCTTCGAGGAGT	TGCAGCCACCACGTCTACTG
SSR2	(CTGATA)5	TGGAAGATGGTTGAAGCAAAGGGT	AGGCATCCATGTCAGATTGCA
SSR3	(GAACTG)5	TGATGCTGCGTACGGCTCAC	CTTCCTGACGCAGAGCGGAG
SSR5	(AGCTGG)6	TCCCCACCAGACTCTGCACC	CAGTGCCAGTTCCAGTGCCA
SSR6	(CACTAC)5	GAAATCCTTCCACCTGGG	CAACAACTTTGCCTGCTT
SSR8	(AGATT)7	GAGAATGGCACAATGAGA	TAGAGGTGGAGCGGATTA
SSR9	(CATAG)5	TTTATACTTCTTCGCCATCA	GGTCAGTTTCCAGGGTGC
SSR12	(CTGGG)5	TGGAGCATCTTTGTTGAG	GGTCGGATAGTAAATAGGG
SSR14	(ATCCA)5	CGCCACAATCTAACTGCT	TCCACCTCATTAACCCTC
SSR17	(ACCAT)5	CACTGACCTCTGCGACTA	GTAAGAATGCTCCCGACA
SSR18	(AGATT)7	GGGCATCGCAGAGTAACAG	GCCGTCCCATACCCAAGT
SSR21	(TGCT)5	GGCTGCCTCATAGACCAC	GAAAGTTACAAACCGACA
SSR24	(TGAT)5	CAGATAAGATTCTTCCGCACTA	ATGGGAAATGGAGGCTGT
SSR26	(GATA)5	TGTGATGAAGCACCCTGAA	GAGCGGTATAAGTTGTCTGG
SSR27	(TGTC)5	TTTTCTTACAGGCTTTATTGCG	ACTCAGCGGTTGGTTTGC
SSR28	(TATT)5	ACGTTATGTTGGGTGCTA	TTGTTCGGTACAGAGGAG
SSR29	(TGTT)5	ATACGCCCAATTAAGGAG	GTCGGTGATGGTTAGCAG
SSR30	(GATA)5	TGCCTGTGATGAAGCACCCTG	AATTTGCCGCGGTTGTGCTG
SSR31	(GATA)5	CAGAGACGGCCTGCAGATGG	GGAAGTGTTGGCACTTGCGT
SSR32	(TTCT)5	GTCACCGAACTGCCCAACCA	GTCTCGCCTCGGCTTCTGAC
SSR34	(ACCA)5	GTACCACCATCGGCCTGTCG	GGGCGTTGTCCGGATGAGAG
SSR36	(TATT)5	AGCGACTTCTGCTGTGTGGT	TGGATGTTCGTGGATGCCCT
SSR38	(TCAT)5	CCTCAGACAATCACTGGCGCT	GGCAGAATCAATCGTCGTGCG
SSR39	(CATC)5	GCCAAAGAGTGGGAGTCGGG	CAAGCGTTGGGCGTGGAGTA
SSR46	(TCCT)5	GATCGGCGGACAAAGAAGGA	TTTCACCTGCCTCTTGCCTT
SSR47	(TCCT)5	TGCACGAGCCATACAGAGACG	ACAGTGGGAACAAGGATGGCG
SSR48	(GATA)5	CGCTGACCGGCCTTTCAAGA	AGTCCTGCTTCCCCGGGATT

**Table 3:** Polymorphism analysis of SSR primers at 8 loci.

Primer	Na	Ne	I	Ho	He	PIC
ssr1	5.000	2.183	1.105	0.244	0.542	0.512
ssr2	7.000	2.730	1.311	0.079	0.634	0.595
ssr3	6.000	3.645	1.460	0.127	0.726	0.684
ssr18	10.000	4.724	1.814	0.449	0.788	0.762
ssr30	4.000	2.363	1.062	0.423	0.577	0.523
ssr32	15.000	3.928	1.786	0.229	0.745	0.711
ssr34	7.000	1.977	1.021	0.171	0.494	0.460
ssr39	15.000	3.248	1.723	0.161	0.692	0.670
Mean	8.625	3.100	1.410	0.235	0.650	0.614

**Table 4:** Genetic diversity analysis of *Passiflora* species and their cultivars.

Species (Cultivars)	*Na*	*Ne*	*I*	*Ho*	*He*	Number
*P. altebilobata*	1.250	1.250	0.260	0.375	0.188	1
*P. edulis*	2.750	1.889	0.701	0.270	0.436	20
*P. edulis* f. *flavicarpa*	2.375	1.642	0.556	0.174	0.326	8
*P.trifasciata*	1.375	1.375	0.347	0.500	0.250	1
*P. wilsonii*	1.000	1.000	0.173	0.250	0.125	1
*P. xishuangbannaensis*	0.750	0.750	0.087	0.125	0.063	1
*P.*’*Lady Margaret*’	1.875	1.783	0.547	0.563	0.375	2
*P. amethystina*	1.375	1.325	0.244	0.313	0.172	2
*P. caerulea*	1.125	1.125	0.087	0.125	0.063	1
*P. incarnata × P. laurifiolia*	2.375	1.880	0.666	0.406	0.410	4
*P. foetida*	1.875	1.700	0.462	0.169	0.290	5
*P. incarnata*	1.500	1.500	0.347	0.375	0.250	2
*P. ligularis*	2.000	1.382	0.436	0.117	0.269	5
*P. miniata*	1.500	1.079	0.113	0.063	0.055	10
*P. miniata* Xishuangbanna Red	0.875	0.875	0.000	0.000	0.000	1
*P. miniata × P. serrulata*	1.375	1.350	0.253	0.333	0.181	6
*P. morifolia*	1.500	1.371	0.414	0.188	0.262	3
*P. quadrangularis*	1.375	1.196	0.192	0.083	0.125	3
*P. serrulata*	1.000	1.000	0.000	0.000	0.000	1
*P. suberosa*	2.125	1.791	0.621	0.390	0.388	6
*P. violacea* ‘*victoria*’	1.250	1.250	0.173	0.250	0.125	2
*P. yucatanensis*	1.250	1.167	0.260	0.188	0.156	2

**Table 5:** Distribution of the Q values of the nine groups.

Group	Number of Varieties Group	Number of Varieties			
		Q < 0.6	Q ≥ 0.6	Q ≥ 0.8	Q ≥ 0.9
1	14	1	13	13	12
2	5	0	5	5	4
3	5	0	5	5	4
4	5	0	5	4	4
5	12	4	8	5	3
6	3	0	3	0	0
7	20	2	18	17	16
8	16	1	15	2	0
9	7	0	7	6	6
Total	87	8	79	57	49

**Table 6:** Letter codes for primer fragment sizes.

Primer Name	Code	Size (bp)	Primer Name	Code	Size (bp)	Primer Name	Code	Size (bp)
SSR1	87	A		366	F		224	O
	93	B		367	G	SSR34	130	A
	99	C		372	H		198	B
	105	D		377	I		202	C
	117	E		387	J		206	D
SSR2	175	A	SSR30	248	A		209	E
	193	B		252	B		210	F
	199	C		256	C		322	G
	203	D		260	D	SSR39	239	A
	205	E	SSR32	144	A		243	B
	211	F		156	B		251	C
	307	G		160	C		259	D
SSR3	220	A		164	D		267	E
	226	B		168	E		275	F
	232	C		172	F		279	G
	238	D		176	G		283	H
	244	E		180	H		287	I
	250	F		184	I		291	J
SSR18	305	A		188	J		295	K
	342	B		192	K		299	L
	347	C		196	L		307	M
	357	D		204	M		315	N
	362	E		212	N		319	O

**Table 7:** Fingerprint profiles of 87 germplasm accessions of the genus *Passiflora*.

Sample	Species (Cultivars)	SSR Fingerprinting
1-*P. altebilobata*	*P. altebilobata*	BICCACBBFL00CCDD
2-*P. edulis*	*P. edulis*	IIDDJLCFLL00CCDD
3-*P. edulis*	*P. edulis*	II00JJCCLLCCCCDD
4-*P. edulis*	*P. edulis*	BIDDJJCCLLACCCDD
5-*P. edulis*	*P. edulis*	IIDDJJCCLLCCCCDD
6-*P. edulis*	*P. edulis*	IIDDJLCCLLCCCCDD
7-*P. edulis* “gui han yi hao” (seeding)	*P. edulis*	GIDFJL00LLCDCEFF
8-*P. edulis* “gui han yi hao” (seeding)	*P. edulis*	IIFFJJFFLLCDEEFF
9-*P. edulis* “gui han yi hao” (seeding)	*P. edulis*	GIDDJJFFLLCDCCFF
10-*P. edulis* “hei mei ren”	*P. edulis*	GIFFJJFFLLDDEEFF
11-*P. edulis* “man tian xing”	*P. edulis*	IIDDJJFFLLAACCDD
12-*P. edulis* “man tian xing”	*P. edulis*	IIDDJJFFLLAACCDD
13-*P. edulis* “tai nong”	*P. edulis*	00DDJLFFLLCDCEDF
14-*P. edulis* “tai nong”	*P. edulis*	GIDDJJFFLLCDCEDF
15-*P. edulis* “tai nong”	*P. edulis*	GIDDJJFFLLCDCEDF
16-*P. edulis* “zi xiang”	*P. edulis*	GIDFIIFFKKCDACDF
17-*P. edulis* “zi xiang”	*P. edulis*	GIDFJLFFLLCDCEDF
18-*P. edulis* “zi xiang”	*P. edulis*	ABFFJLFFLLCDEEDF
19-*P. edulis* “gui han yi hao”	*P. edulis*	GGFFJJFFLLDDEEFF
20-*P. edulis* “gui han yi hao”	*P. edulis*	FFFFJJFFLLDDEEFF
21-*P. edulis* “gui han yi hao”	*P. edulis*	GGFFJJCFLLDDEEFF
22-*P. edulis* f. *flavicarpa* “16#”	*P. edulis* f. *flavicarpa*	BIDDJJCCLLCCCCDD
23-*P. edulis* f. *flavicarpa* “6#”	*P. edulis* f. *flavicarpa*	BBDDJJCCLLACACDD
24-*P. edulis* f. *flavicarpa* “mi zhuan”	*P. edulis* f. *flavicarpa*	EECCIICCMMCCACDF
25-*P. edulis* f. *flavicarpa* “qing ren guo”	*P. edulis* f. *flavicarpa*	BBDDLLCCLLCCCCDD
26-*P. edulis* f. *flavicarpa* (stock)	*P. edulis* f. *flavicarpa*	BCEEJJCCLLAACCDD
27-*P. edulis* f. *flavicarpa*”9# “	*P. edulis* f. *flavicarpa*	BBDDJJCCLLACCCDD
28-*P. edulis* f. *flavicarpa*	*P. edulis* f. *flavicarpa*	IIDDJLCCLLACCC00
29-*P. edulis* f. *flavicarpa*	*P. edulis* f. *flavicarpa*	IIDDJLCCLLACCCDD
30-*P. trifasciata*	*P.trifasciata*	GI00IJACCDCCCCDD
31-*P. wilsonii*	*P. wilsonii*	BI00IJBBLL00CCDD
32-*P. xishuangbannaensis*	*P. xishuangbannaensis*	BH0000BBLL00CCDD
33-*P*. ‘Lady Margaret’	*P.* ‘Lady Margaret’	BIACNNCCHLCCACCC
34-*P*. ‘Lady Margaret’	*P.* ‘Lady Margaret’	BGACINCCHLCCACDD
35-*P. amethystina*	*P. amethystina*	DGBBGGBBIIDDABAD
36-*P. amethystina*	*P. amethystina*	DGBBGGBBIIDDABAA
37-*P. caerulea*	*P. caerulea*	DDBBIJBBJJDDBBDD
38-*P. incarnata* × *P. laurifiolia*	*P. incarnata × P. laurifiolia*	BGACMMEELLBCAA00
39-*P. incarnata* × *P. laurifiolia*	*P. incarnata × P. laurifiolia*	EECEIICCLLBDABCC
40-*P. incarnata* × *P. laurifiolia*	*P. incarnata × P. laurifiolia*	EECEOOCCLLBDABCC
41-*P. incarnata* × *P. laurifiolia*	*P. incarnata × P. laurifiolia*	EECEOOCCELBDABCC
42-*P. foetida*	*P. foetida*	00BBHHDDNNCCBB00
43-*P. foetida*	*P. foetida*	AIBBHHCCNNCCBB00
44-*P. foetida*	*P. foetida*	AABBHH00NNCDBBDD
45-*P. foetida*	*P. foetida*	GIBBHHGGLNCCBBFF
46-*P. foetida*	*P. foetida*	GIBBHH00NNCDBB00
47-*P. incarnata*	*P. incarnata*	EECC00EEOOBCACCC
48-*P. incarnata*	*P. incarnata*	BBCCHMEEOOBCACCC
49-*P. ligularis*	*P. ligularis*	IICCII00MMCCCC00
50-*P. ligularis*	*P. ligularis*	IICCIICCMMCCCCDD
51-*P. ligularis*	*P. ligularis*	IICCIICCMMCCCC00
52-*P. ligularis*	*P. ligularis*	IICCIICCMMCCCCDD
53-*P. ligularis*	*P. ligularis*	GIDDJJFFLLCDCEDE
54-*P. miniata*	*P. miniata*	BICCIICCEHCCCCDD
55-*P. miniata*	*P. miniata*	BICCIICCHHCCCC00
56-*P. miniata*	*P. miniata*	BJCCIICCHHCCCC00
57-*P. miniata*	*P. miniata*	BBCCIICCHHCCCC00
58-*P. miniata*	*P. miniata*	BBCCIICCHHCCCCDD
59-*P. miniata*	*P. miniata*	BBCCIICCHHCCCCDD
60-*P. miniata*	*P. miniata*	BBCCIICCHHCCCCDD
61-*P. miniata*	*P. miniata*	BBCCIICCHHCCCC00
62-*P. miniata*	*P. miniata*	BGCCIICCHHCCCC00
63-*P. miniata*	*P. miniata*	BBCCIICCHHCCCCDD
64-*P. miniata* Xishuangbanna Red	*P. miniata* Xishuangbanna Red	00CCIICCHHCCCCDD
65-*P. miniata* × *P. serrulata*	*P. miniata × P. serrulata*	BHCCIICCHLCDCCDD
66-*P. miniata* × *P. serrulata*	*P. miniata × P. serrulata*	BHCCIICCHLCDCCDD
67-*P. miniata* × *P. serrulata*	*P. miniata × P. serrulata*	BHCCIICCHLCDCCDD
68-*P. miniata* × *P. serrulata*	*P. miniata × P. serrulata*	HHCCIICCHLCDCCDD
69-*P. miniata* × *P. serrulata*	*P. miniata × P. serrulata*	HHCCIICCHLCDCCDD
70-*P. miniata* × *P. serrulata*	*P. miniata × P. serrulata*	BHCCIICCHLCDCCDD
71-*P. morifolia*	*P. morifolia*	0000DJBBAACDCCDD
72-*P. morifolia*	*P. morifolia*	0000JJBCLLCCCCDD
73-*P. morifolia*	*P. morifolia*	0000BBBCBB00CCDD
74-*P. quadrangularis*	*P. quadrangularis*	AAFFGGAALLBBDDDD
75-*P. quadrangularis*	*P. quadrangularis*	AAFFGGAALLBBDDDD
76-*P. quadrangularis*	*P. quadrangularis*	AAFFIIAALLBCDDBD
77-*P. serrulata*	*P. serrulata*	HHCCIICCLLDDCCDD
78-*P. suberosa*	*P. suberosa*	II00IJ00GLBBCCDG
79-*P. suberosa*	*P. suberosa*	00000000GG0000DG
80-*P. suberosa*	*P. suberosa*	00000000GG00AC00
81-*P. suberosa*	*P. suberosa*	JJ00IJ00GGCCCC00
87-*P. suberosa*	*P. suberosa*	AI00DIACGG00CC00
83-*P. suberosa*	*P. suberosa*	0000EE00GG00CCGG
84-*P. violacea* ‘victoria’	*P. violacea* ‘victoria’	DI00IIBBLLCCAB00
85-*P. violacea* ‘victoria’	*P. violacea* ‘victoria’	DIBBIIBBLLCCABDD
86-*P. yucatanensis*	*P. yucatanensis*	II00BK00DDCDCC00
87-*P. yucatanensis*	*P. yucatanensis*	II00FF00DDACCCDD

## Data Availability

Data are contained within the article and Supplementary Materials.

## Supplementary Materials

The following supporting information can be downloaded at: https://www.mdpi.com/article/10.3390/ijms251910815/s1.
