# Supplementary material for: Genetic Diversity Analysis and Fingerprint Construction for 87 Passionfruit (Passiflora spp.) Germplasm Accessions on the Basis of SSR Fluorescence Markers
Source: Int J Mol Sci. 2024 Oct 8;25(19):10815. doi: 10.3390/ijms251910815 (PMC11476748; doi:10.3390/ijms251910815)
Supplement: Supplementary file 1 [file ijms-25-10815-s001.zip › ijms-3188154-supplementary.pdf]

Genetic Diversity Analysis and Fingerprint Construction for 87 Passionfruit (*Passiflora* spp.) Germplasms on the Basis of SSR Fluorescence Markers

Fengchan Wu [1], Guojun Cai [2], Peiyu Xi [1], Yulin Guo [1],Meng Xu [3] and Anding Li[1,*]

Table S1. Basic information of test materials

Code	Name of the Variety	Species	origin
1	*P. altebilobata*	*P. altebilobata*	Xishuangbanna
2	*P. edulis*	*P. edulis*	Xishuangbanna
3	*P. edulis*	*P. edulis*	Xishuangbanna
4	*P. edulis*	*P. edulis*	Xishuangbanna
5	*P. edulis*	*P. edulis*	Xishuangbanna
6	*P. edulis*	*P. edulis*	Xishuangbanna
7	*P. edulis "gui han yi hao"*	*P. edulis*	Pingtang
8	*P. edulis "gui han yi hao"*	*P. edulis*	Pingtang
9	*P. edulis "gui han yi hao"*	*P. edulis*	Pingtang
10	*P. edulis "gui han yi hao"(seeding)*	*P. edulis*	Pingtang
11	*P. edulis "gui han yi hao"(seeding)*	*P. edulis*	Pingtang
12	*P. edulis "gui han yi hao"(seeding)*	*P. edulis*	Pingtang
13	*P. edulis "hei mei ren"*	*P. edulis*	Pingtang
14	*P. edulis "man tian xing"*	*P. edulis*	Pingtang
15	*P. edulis "man tian xing"*	*P. edulis*	Pingtang
16	*P. edulis "tai nong"*	*P. edulis*	Xishuangbanna
17	*P. edulis "tai nong"*	*P. edulis*	Pingtang
18	*P. edulis "tai nong"*	*P. edulis*	Pingtang
19	*P. edulis "zi xiang"*	*P. edulis*	Pingtang
20	*P. edulis "zi xiang"*	*P. edulis*	Pingtang
21	*P. edulis "zi xiang"*	*P. edulis*	Pingtang
22	*P. edulis* f. *flavicarpa "16#"*	*P. edulis* f. *flavicarpa*	Xishuangbanna
23	*P. edulis* f. *flavicarpa "6#"*	*P. edulis* f. *flavicarpa*	Pingtang
24	*P. edulis f. flavicarpa "mi zhuan"*	*P. edulis* f. *flavicarpa*	Pingtang
25	*P. edulis* f. *flavicarpa "qing ren guo"*	*P. edulis* f. *flavicarpa*	Pingtang
26	*P. edulis* f. *flavicarpa (stock)*	*P. edulis* f. *flavicarpa*	Pingtang
27	*P. edulis* f. *flavicarpa"9# "*	*P. edulis* f. *flavicarpa*	Pingtang
28	*P. edulis* f. *flavicarpa*	*P. edulis* f. *flavicarpa*	Pingtang
29	*P. edulis* f. *flavicarpa*	*P. edulis* f. *flavicarpa*	Pingtang
30	*P.trifasciata*	*P.trifasciata*	Xishuangbanna
31	*P. wilsonii*	*P. wilsonii*	Xishuangbanna
32	*P. xishuangbannaensis*	*P. xishuangbannaensis*	Xishuangbanna

33	*P.'Lady Margaret'*	*P.'Lady Margaret'*	Pingtang
34	*P.'Lady Margaret'*	*P.'Lady Margaret'*	Pingtang
35	*P. amethystina*	*P. amethystina*	Pingtang
36	*P. amethystina*	*P. amethystina*	Pingtang
37	*P. caerulea*	*P. caerulea*	Xishuangbanna
38	*P. incarnata ×P. laurifiolia*	*P. incarnata ×P. laurifiolia*	Pingtang
39	*P. incarnata ×P. laurifiolia*	*P. incarnata ×P. laurifiolia*	Pingtang
40	*P. incarnata ×P. laurifiolia*	*P. incarnata ×P. laurifiolia*	Pingtang
41	*P. incarnata ×P. laurifiolia*	*P. incarnata ×P. laurifiolia*	Pingtang
42	*P. foetida*	*P. foetida*	Xishuangbanna
43	*P. foetida*	*P. foetida*	Xishuangbanna
44	*P. foetida*	*P. foetida*	Xishuangbanna
45	*P. foetida*	*P. foetida*	Pingtang
46	*P. foetida*	*P. foetida*	Pingtang
47	*P. incarnata*	*P. incarnata*	Pingtang
48	*P. incarnata*	*P. incarnata*	Pingtang
49	*P. ligularis*	*P. ligularis*	Xishuangbanna
50	*P. ligularis*	*P. ligularis*	Xishuangbanna
51	*P. ligularis*	*P. ligularis*	Xishuangbanna
52	*P. ligularis*	*P. ligularis*	Pingtang
53	*P. ligularis*	*P. ligularis*	Pingtang
54	*P. miniata*	*P. miniata*	Xishuangbanna
55	*P. miniata*	*P. miniata*	Xishuangbanna
56	*P. miniata*	*P. miniata*	Xishuangbanna
57	*P. miniata*	*P. miniata*	Xishuangbanna
58	*P. miniata*	*P. miniata*	Xishuangbanna
59	*P. miniata*	*P. miniata*	Xishuangbanna
60	*P. miniata*	*P. miniata*	Xishuangbanna
61	*P. miniata*	*P. miniata*	Pingtang
62	*P. miniata*	*P. miniata*	Pingtang
63	*P. miniata*	*P. miniata*	Xishuangbanna
64	*P. miniata Xishuangbanna Red*	*P. miniata Xishuangbanna Red*	Xishuangbanna
65	*P. miniata× P. serrulata*	*P. miniata× P. serrulata*	Xishuangbanna
66	*P. miniata× P. serrulata*	*P. miniata× P. serrulata*	Xishuangbanna
67	*P. miniata× P. serrulata*	*P. miniata× P. serrulata*	Xishuangbanna
68	*P. miniata× P. serrulata*	*P. miniata× P. serrulata*	Xishuangbanna
69	*P. miniata× P. serrulata*	*P. miniata× P. serrulata*	Xishuangbanna
70	*P. miniata× P. serrulata*	*P. miniata× P. serrulata*	Xishuangbanna
71	*P. morifolia*	*P. morifolia*	Xishuangbanna
72	*P. morifolia*	*P. morifolia*	Xishuangbanna
73	*P. morifolia*	*P. morifolia*	Xishuangbanna
74	*P. quadrangularis*	*P. quadrangularis*	Xishuangbanna
75	*P. quadrangularis*	*P. quadrangularis*	Xishuangbanna
76	*P. quadrangularis*	*P. quadrangularis*	Pingtang

77	*P. serrulata*	*P. serrulata*	Xishuangbanna
78	*P. suberosa*	*P. suberosa*	Xishuangbanna
79	*P. suberosa*	*P. suberosa*	Xishuangbanna
80	*P. suberosa*	*P. suberosa*	Xishuangbanna
81	*P. suberosa*	*P. suberosa*	Xishuangbanna
82	*P. suberosa*	*P. suberosa*	Xishuangbanna
83	*P. suberosa*	*P. suberosa*	Xishuangbanna
84	*P. violacea 'victoria'*	*P. violacea 'victoria'*	Pingtang
85	*P. violacea 'victoria'*	*P. violacea 'victoria'*	Pingtang
86	*P. yucatanensis*	*P. yucatanensis*	Xishuangbanna
87	*P. yucatanensis*	*P. yucatanensis*	Xishuangbanna

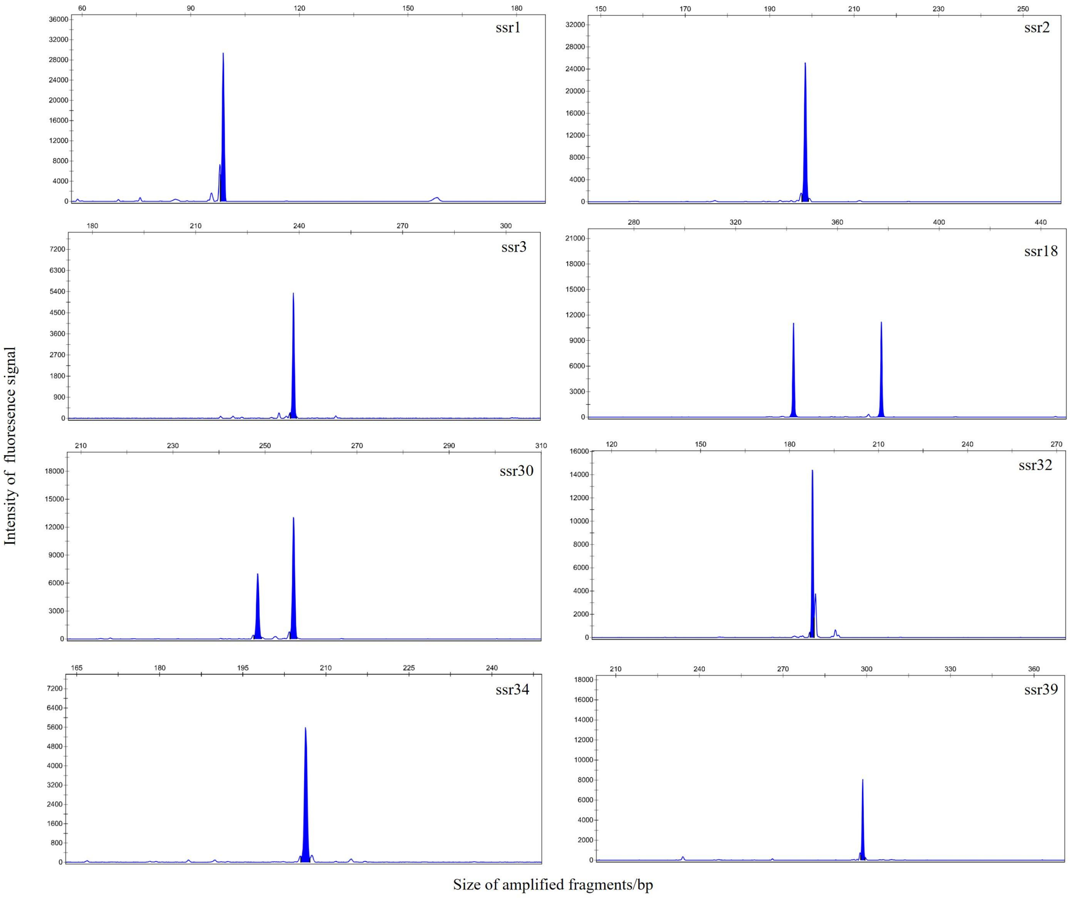

Figuer S1. Amplification of 4-P. edulis samples using 8 core primer

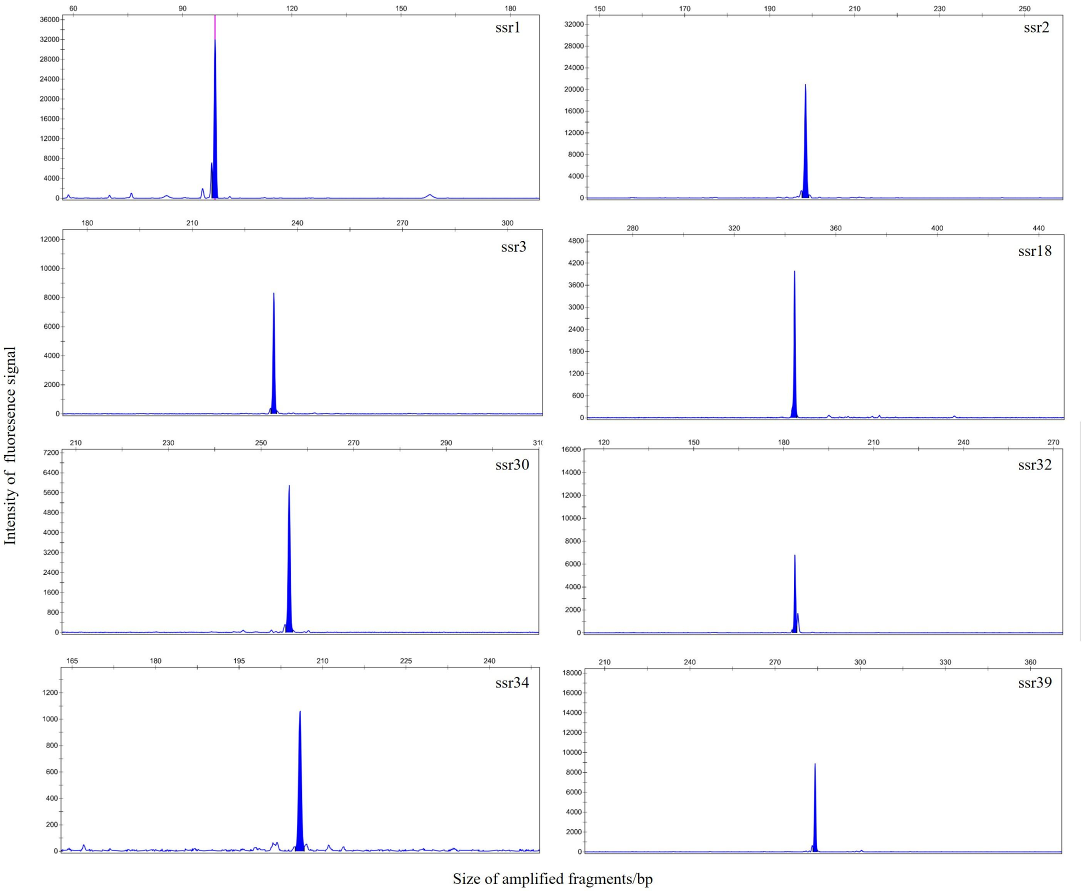

Figuer S2. Amplification of 58-P. miniata samples using 8 core primer
